# One step hydrothermal synthesis of magnetically separable rGO supported Fe₃O₄ and Ag nanoparticles for adsorption and reduction of organic pollutants

**DOI:** 10.1038/s41598-025-12170-9

**Published:** 2025-07-27

**Authors:** Eman F. Aboelfetoh

**Affiliations:** Chemistry Department, Faculty of Science, Tanta University, Tanta, 31527 Egypt

**Keywords:** Reduced graphene oxide, Error analysis, Ternary nanocomposite, Methyl violet adsorption, Magnetic separation, Catalytic reduction, Catalysis, Environmental chemistry, Physical chemistry

## Abstract

The development of efficient adsorbents and catalysts is crucial for enhanced pollutant removal and catalytic performance. In this study, a magnetically separable rGO/Fe_3_O_4_/Ag nanocomposite was synthesized via a facile one step hydrothermal method, enabling simultaneous reduction of graphene oxide (GO) and in situ deposition of Fe_3_O_4_ and Ag nanoparticles. The nanocomposite’s structure, surface features, and magnetic properties were confirmed through appropriate characterization techniques. The nanocomposite exhibited high adsorption efficiency toward methyl violet 2B (MV), with performance evaluated across varying dye concentrations, pH, temperature, and adsorbent dosages. Statistical error analysis (reduced χ^2^, RMSE, SSE) validated the applicability of a pseudo-second-order kinetics. The adsorption data also fit Langmuir isotherm, revealing a maximum uptake (q_max_) of 168.70 mg/g. Employing NaBH_4_ as reductant, the nanocomposite achieved rapid *p*-nitroaniline (*p*-NA) hydrogenation to *p*-phenylenediamine (*p*-PDA), achieving 97.60% conversion within 3 min and a rate constant of 0.95 min⁻^1^, consistent with pseudo-first-order kinetics. The nanocomposite’s strong magnetic responsiveness (Ms = 30 emu/g) enabled efficient separation and reusability, maintaining stable performance over five adsorption and eight catalytic cycles.

## Introduction

Organic dyes released by various industries cause significant environmental pollution. Many synthetic textile dyes are mutagenic and carcinogenic, leading to detrimental effects on aquatic ecosystems, such as species extinction and reduced photosynthetic activity^1–3^. A variety of techniques have been employed to treat dye-containing wastewater, a prevalent pollutant in industrial effluents, particularly from textile industries. Among these method^4–8^, the adsorption technique stands out as the most effective for treating large volumes of high-strength wastewater. This method is favored for its cost-effectiveness, low energy demand, and ability to enable adsorbent regeneration, making it highly suitable for sustainable and scalable wastewater treatment^9,10^. The hydrogenation of nitroarenes in water is crucial for environmental protection and industrial production. Nitroaromatic compounds, discharged from industrial and agricultural sources, are toxic and pose serious risks to ecosystems and human health^11^. Their reduction to aromatic amines not only detoxifies wastewater but also yields valuable intermediates for the synthesis of pharmaceuticals, dyes, plastics, and pesticides^12,13^. This process is therefore vital for both pollution control and the production of high-value chemicals. To address both dye adsorption and nitroarene reduction, nanocomposites with tailored multifunctional properties are highly desirable. Magnetite (Fe_3_O_4_) nanoparticles are widely recognized for their redox activity, catalytic potential, and magnetic properties, allowing for facile separation from treated water. However, their practical utility is limited by a tendency to agglomerate and oxidize, which reduces surface reactivity and catalytic performance^14^.

Reduced graphene oxide (rGO) serves as an exceptional support material for Fe_3_O_4_ nanoparticles, significantly enhancing their catalytic and adsorption capabilities. With its large surface area, π-conjugated structure, and excellent electrical conductivity, rGO effectively stabilizes Fe_3_O_4_ nanoparticles, preventing aggregation while promoting rapid electron transfer—key factors in boosting catalytic efficiency^15^. This combination improves both adsorption and catalytic performance, making it highly suitable for multifunctional environmental applications.

To further improve catalytic performance, silver nanoparticles (Ag NPs) have been incorporated into the Fe_3_O_4_/rGO composite. Ag is known for its outstanding catalytic efficiency in redox reactions, particularly the reduction of nitro compounds, as well as for its antimicrobial activity and strong surface plasmon resonance^12,16^. Ag NPs provide a cost-efficient alternative to Au, Pt, or Pd nanoparticles, making it suitable for large-scale applications^17^. The integration of rGO’s structural advantages, Fe_3_O_4_’s magnetic responsiveness, and Ag’s catalytic properties offers a robust platform for advanced environmental remediation. Although binary nanocomposites including rGO/Fe_3_O_4_, rGO/Ag, and Fe_3_O_4_/Ag have been extensively investigated^18–20^, ternary rGO/Fe_3_O_4_/Ag nanocomposites remain underexplored, particularly those synthesized via a simple, one-pot method. Most reported approaches rely on multi-step processes or require external stabilizers, increasing both production costs and process complexity^21–23^. This study presents an innovative solution through a single-step hydrothermal synthesis of rGO/Fe_3_O_4_/Ag nanocomposite, wherein GO reduction and the in situ formation of Fe_3_O_4_ and Ag NPs occur simultaneously. The resulting material combines rGO’s high adsorption capacity, Fe_3_O_4_’s magnetic separability, and Ag’s redox efficiency, demonstrating exceptional performance in methyl violet (MV) dye removal and catalytic reduction of *p*-nitroaniline (*p*-NA). The method is cost-effective, scalable, and eco-friendly, offering a promising route for sustainable water treatment applications.

## Materials and methods

### Materials

All chemicals employed in this investigation were of analytical grade, purchased from established commercial suppliers. The materials consisted of graphite powder (Jodex, UK) along with various analytical-grade compounds: polyethylene glycol (PEG), 99% pure ferrous sulfate, 85% phosphoric acid, silver nitrate, 98% sulfuric acid, potassium permanganate, absolute ethanol (≥ 99% purity), 37% hydrochloric acid, and 30% hydrogen peroxide solution. With the exception of graphite, all chemical substances were supplied by Fluka AG, Switzerland. Methyl violet 2B (C_24_H_28_ClN_3_), mol. wt. = 393.95 g/mol was acquired from Sigma-Aldrich chemicals. Sodium borohydride (NaBH_4_) and *p*-nitroaniline (C_6_H_6_N_2_O_2_) were acquired from Merck, Germany. All chemicals were used as received. Working solutions were prepared following standard methods using distilled H_2_O.

### Material synthesis

#### Synthesis of graphene oxide (GO)

Graphene oxide was synthesized using a modified Hummers’ method. Briefly, graphite powder (1 g) was uniformly mixed with a concentrated acid solution comprising sulfuric acid (108 mL) and phosphoric acid (27 mL). The mixture was cooled in an ice bath to control the reaction temperature, and 6 g of KMnO_4_ was slowly added under continuous stirring to ensure uniform oxidation. The reaction was maintained at room temperature for 72 h, during which the color gradually changed from dark purplish green to dark brown, confirming the advancement of the oxidation process. To stop the reaction, 20 mL of H_2_O_2_ was added, turning the mixture bright yellow due to the reduction of manganese species. Residual salts were removed by treating the mixture with 10% HCl. Washing with distilled water was carried out by decantation until the supernatant reached neutral pH. During this step, exfoliation of graphite oxide occurred, forming a thick graphene oxide (GO) gel. The resulting gel was oven-dried at 70 °C overnight, yielding the final material.

#### One step hydrothermal synthesis of rGO/Fe_3_O_4_/Ag nanocomposite

**Scheme 1:**
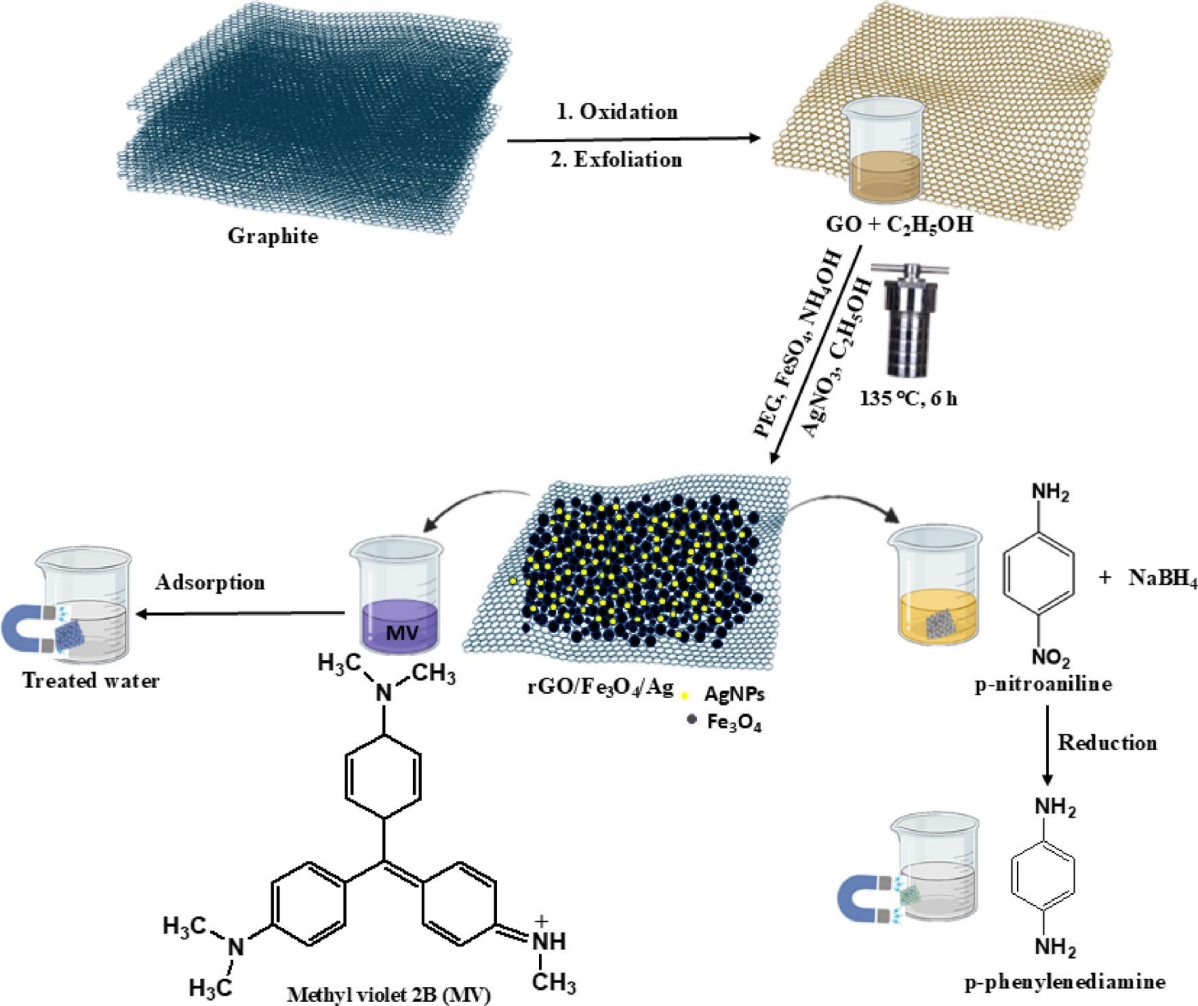
Synthesis steps for rGO/Fe_3_O_4_/Ag nanocomposite, illustrating its application in the adsorption of MV dye and the catalytic reduction of *p*-nitroaniline in the presence of NaBH_4_.

The ternary nanocomposite was synthesized via a one step hydrothermal process that simultaneously facilitated the reduction of GO to rGO, the in situ formation of Fe_3_O_4_ NPs, and the uniform decoration of Ag NPs on the rGO sheets (Scheme 1). The reaction was initiated by dissolving PEG (0.1 g) in 20 mL of a 0.025 molar FeSO_4_ aqueous solution. The mixture was then treated with 1.3 mL of 28% NH_4_OH solution under continuous stirring (200 rpm) for 45 min. Next, 10 mL 0.002 M AgNO_3_ and pre-sonicated suspension of GO (0.5 g GO in 20 mL H_2_O + 10 mL ethanol) were added, plus 10 mL additional ethanol. The reaction mixture was sealed in a stainless-steel autoclave with a Teflon liner and exposed to hydrothermal conditions (135 °C, 6 h). Upon reaching room temperature, the product was sequentially rinsed with distilled H_2_O and oven-dried. The binary rGO/Fe_3_O_4_ nanocomposite was synthesized following the same procedure but without AgNO_3_. For comparison, reduced graphene oxide (rGO) was also prepared under identical conditions, omitting both AgNO_3_ and FeSO_4_.

The synthesized samples were characterized for their crystallinity and structural properties by X-ray diffraction (XRD; GNR APD 2000 PRO system). Molecular functional groups were analyzed by Fourier transform infrared spectroscopy (FTIR) using a JASCO 4100 in transmission mode (400–4000 cm^−1^) with KBr-diluted samples. Magnetic hysteresis measurements were acquired at room temperature using a Lake Shore 7410 vibrating sample magnetometer (VSM). Transmission electron microscopy (TEM, JEOL JEM-2100) was employed to examine the size and distribution of Ag and Fe_3_O_4_ nanoparticles (NPs) in the rGO/Fe_3_O_4_/Ag nanocomposite. Morphological analysis and elemental distribution were investigated using a JEOL JSM-IT100LA scanning electron microscope (SEM) equipped with energy-dispersive X-ray spectroscopy (EDX). Surface chemical states were probed by X-ray photoelectron spectroscopy (Thermo Fisher K-ALPHA XPS) with Al K-α radiation (400 µm spot size, 10^−9^ mbar), acquiring survey scans at 200 eV pass energy and high-resolution regions at 50 eV. Optical properties were assessed by UV–vis spectroscopy (Cary Bio100). The surface area and porosity of the samples (rGO, rGO/Fe_3_O_4_, and rGO/Fe_3_O_4_/Ag) were evaluated using nitrogen adsorption–desorption isotherms at 77 K, employing the Brunauer–Emmett–Teller (BET) method for surface area calculations and the Barrett–Joyner–Halenda (BJH) method for pore size distribution. Prior to analysis, all samples were degassed under vacuum at 110 °C for 6 h.

### Adsorption experiments

The batch-mode adsorption investigations employed a shaking water bath set to the desired temperature, maintaining the process until equilibrium was reached. Most trials were conducted under dark conditions with a 2-h contact period. A defined quantity of magnetic nanocomposite (rGO/Fe_3_O_4_/Ag) was introduced into 25 mL aliquots of MV dye solution at varying initial concentrations (8–150 mg/L), followed by agitation at 130 rpm. After a certain time, the nanocomposite was magnetically collected, and the residual MV concentration was quantified utilizing a UV–VIS spectrophotometer. The following formula was used to calculate the dye removal efficiency:

1 $$\text{Removal }\left(\%\right)=\frac{{\text{C}}_{\text{o}}-{\text{C}}_{\text{t}}}{{\text{C}}_{0}} \times 100$$

where, C_0_ (mg/L) signifies the initial dye concentration, while C_t_ (mg/L) corresponds to the measured concentration after contact time t.

The dye’s adsorption capacity at equilibrium was determined as follow.

2 $${q}_{e}=\frac{\left({C}_{0}-{C}_{e}\right) V}{m}$$

The parameter q_e_ (mg/g) denotes the nanocomposite’s equilibrium adsorption capacity, while C_e_ (mg/L) indicates the dye concentration at equilibrium. The volume of simulated wastewater is given as V (L), and the dose of adsorbent is given as m (g).

### Catalytic evaluation

The catalytic capability of the ternary nanocomposite (rGO/Fe_3_O_4_/Ag) was assessed in the NaBH_4_-assisted reduction of *p*-NA (*p*-nitroaniline) to *p*-PDA (*p*-phenylenediamine). The reaction mixture was established with 10 mL of aqueous solution containing *p*-NA (0.1 mM) and NaBH_4_ (8 mM). The reduction process was initiated by adding 0.005 g of nanocomposite, as evidenced by the fading of the solution’s yellow color. The reduction kinetics were monitored via UV–visible spectroscopy, quantifying the temporal decrease in characteristic *p*-NA absorption at λ_max_ = 380 nm. After each cycle, the catalyst was magnetically separated, washed, and dried, ensuring its reusability in further reactions. The reduction efficiency was assessed as follows

3 $$\text{Reduction }\left(\%\right)=\frac{{\text{A}}_{\text{o}}-{A}_{\text{t}}}{{\text{A}}_{0}} \times 100$$

A_0_ represents the initial absorbance of *p*-NA, while A_t_ denotes the measured absorbance at reaction time t.

## Results and discussion

### Characterization of nanocomposite

Multiple analytical techniques were utilized to characterize the structural, morphological, magnetic, and electronic properties of the synthesized materials.

#### XRD analysis

**Fig. 1:**
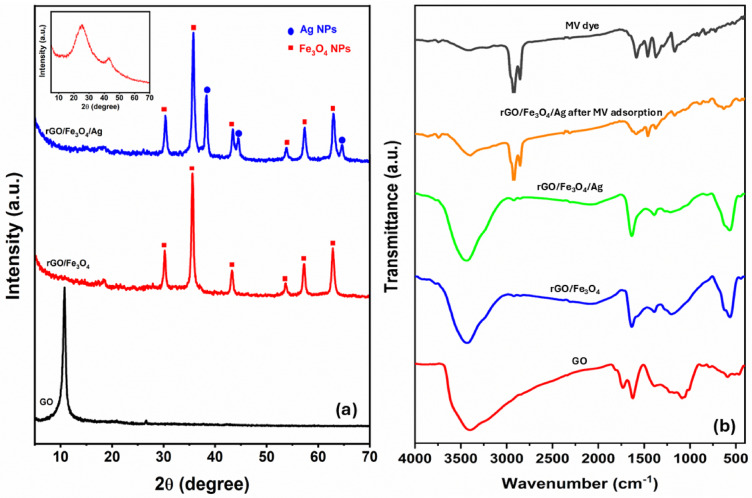
(**a**) XRD patterns of the synthesized materials with inset showing rGO; (**b**) FTIR spectra of the synthesized materials, pure MV dye and rGO/Fe_3_O_4_/Ag nanocomposite after MV adsorption.

The phase composition of GO and other synthesized samples was investigated using XRD. As proven in Fig. 1a, GO displays a definite sharp peak at 2θ = 10.74°, which coincides with the (001) plane^24,25^, showing that graphite has successfully oxidized and generated graphite oxide rich in oxygen-containing groups. The rGO/Fe_3_O_4_ nanocomposite shows all characteristic reflections of phase-pure magnetite (JCPDS 85-1436), with prominent peaks at 2θ = 30.22° (220), 35.56° (311), 43.27° (400), 53.65° (422), 57.25° (511), and 62.83° (440)^26^. These diffraction features confirm the preservation of the inverse spinel configuration within the nanocomposite. In addition to the Fe_3_O_4_ peaks already noted, the rGO/Fe_3_O_4_/Ag nanocomposites’ XRD pattern also showed new peaks at 2θ = 38.18°, 44.65°, and 64.77°. These peaks correspond to the (111), (200), and (220) planes of FCC lattice of AgNPs (JCPDS 04-0784))^27,28^. For both nanocomposites, the absence of distinct rGO signals in the XRD pattern is likely due to the intercalation of Fe_3_O_4_ and Ag nanoparticles (NPs) between the rGO layers, which disrupts the regular structure of rGO^29,30^. Additionally, the strong signals from Ag and Fe_3_O_4_ NPs may overshadow the weaker rGO peaks in the XRD pattern^30,31^. In the absence of FeSO_4_ and AgNO_3_, the XRD pattern (inset of Fig. 1) confirmed the reduction of GO to rGO, showing characteristic peaks at 2θ = 25.55° and 43.01°^19,32^. The mean crystallite sizes of Fe_3_O_4_ and Ag nanostructures were estimated via the Debye–Scherrer equation (Eq. 4)^33,34^, applied to the most intense diffraction peaks corresponding to each crystalline phase in the nanocomposite.

4 $$D = \frac{0.9 \lambda }{{\beta \cos \theta }}$$

In this equation, D is crystallite diameter (nm), λ is X-ray wavelength (0.15406 nm, Cu-Kα), K is the shape factor (0.9), β is FWHM (radians), and θ is Bragg angle (radians). The average crystallite size (D) of Fe_3_O_4_ NPs was approximately 15.75 nm in both the rGO/Fe_3_O_4_ and rGO/Fe_3_O_4_/Ag nanocomposites, suggesting that Ag loading did not significantly alter the Fe_3_O_4_ crystal structure. In contrast, the Ag NPs in the rGO/Fe_3_O_4_/Ag composite exhibited a larger crystallite size of approximately 18.68 nm.

#### FTIR analysis

FTIR analysis (Fig. 1b) demonstrated the chemical evolution from GO to nanocomposites: GO exhibited characteristic bands at 3420 cm^−1^ (O–H stretch), 1726 cm^−1^ (C=O, carboxyl), 1628 cm^−1^ (C=C, sp^2^ carbon), 1225 cm^−1^ (C–OH bend), and 1075 cm^−1^ (C–O stretch), while rGO/Fe_3_O_4_ showed complete disappearance of carboxyl groups (1726 cm^−1^), significant reduction of oxygen functionalities, and new Fe–O vibrations (620–570 cm^−1^)^35^, confirming successful GO reduction and Fe_3_O_4_ incorporation in the nanocomposite structure. The rGO/Fe_3_O_4_/Ag spectrum showed similar features to rGO/Fe_3_O_4_, with both exhibiting the characteristic Fe–O vibration band (620–570 cm^−1^)^35^. The absence of the C=O stretching vibration at 1726 cm^−1^, along with the noticeable weakening of other oxygen-related absorption bands, indicates a successful reduction of GO to rGO in both composites.

#### VSM analysis

**Fig. 2:**
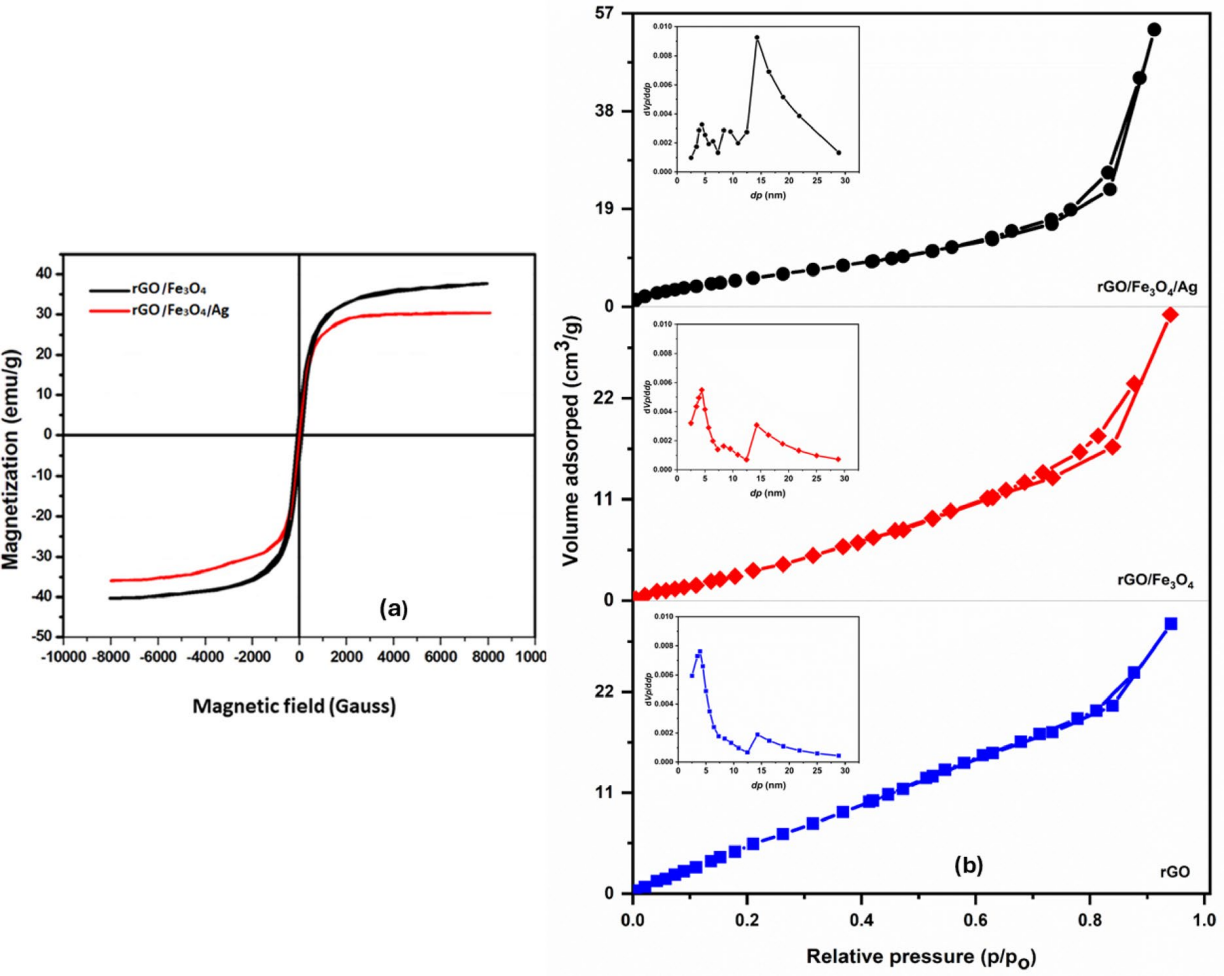
(**a**) VSM curves of synthesized nanocomposites and (**b**) N_2_ adsorption–desorption isotherms of rGO, rGO/Fe_3_O_4_, and rGO/Fe_3_O_4_/Ag nanocomposites, with corresponding BJH pore size distribution curves shown in the inset.

The magnetic characteristics of rGO/Fe_3_O_4_ and rGO/Fe_3_O_4_/Ag nanocomposites were analyzed via VSM at ambient temperature (Fig. 2a). The rGO/Fe_3_O_4_/Ag nanocomposite exhibited a saturation magnetization (Ms) of 30 emu/g, lower than that of rGO/Fe_3_O_4_ (35 emu/g), attributed to the nonmagnetic Ag NPs layer on the surface. The M–H curves displayed negligible remanence (Mr) and coercivity (Hc), confirming the superparamagnetic behavior of both nanocomposites^32^. Despite the absence of hysteresis, the nanocomposite shows a relatively high saturation magnetization of 30 emu/g, which allows for rapid and efficient separation under an external magnetic field (Scheme 1). This magnetic behavior is typical of Fe_3_O_4_ nanoparticles below 20 nm^36^, which respond strongly to external fields but do not retain magnetization once the field is removed^37^. This prevents particle aggregation and allows for easy redispersion, making the rGO/Fe_3_O_4_/Ag nanocomposite both magnetically separable and recyclable—a significant advantage for practical applications.

#### Surface characteristics

The specific surface area and pore structure of rGO, rGO/Fe_3_O_4_, and rGO/Fe_3_O_4_/Ag were evaluated using BET analysis. The N_2_ adsorption–desorption isotherms for all samples are presented in Fig. 2b. The rGO/Fe_3_O_4_/Ag nanocomposite exhibited the highest surface area at 39.5 m^2^/g, compared to 31.0 m^2^/g for rGO/Fe_3_O_4_ and 25.6 m^2^/g for rGO. Similarly, the total pore volume increased from 0.0455 cm^3^/g (rGO) to 0.048 cm^3^/g (rGO/Fe_3_O_4_), reaching 0.085 cm^3^/g for rGO/Fe_3_O_4_/Ag nanocomposite, indicating a substantial enhancement in porosity upon incorporation of Fe_3_O_4_ and Ag NPs. The insets in Fig. 2b show the pore size distributions derived from BJH analysis. The rGO/Fe_3_O_4_/Ag nanocomposite exhibits a broad pore size range from 2.38 to 28.86 nm, with an average pore diameter of 14.27 nm. This wide distribution of mesopores enhances dye diffusion and adsorption, rendering rGO/Fe_3_O_4_/Ag a more efficient adsorbent compared to its individual components.

#### XPS analysis

**Fig. 3:**
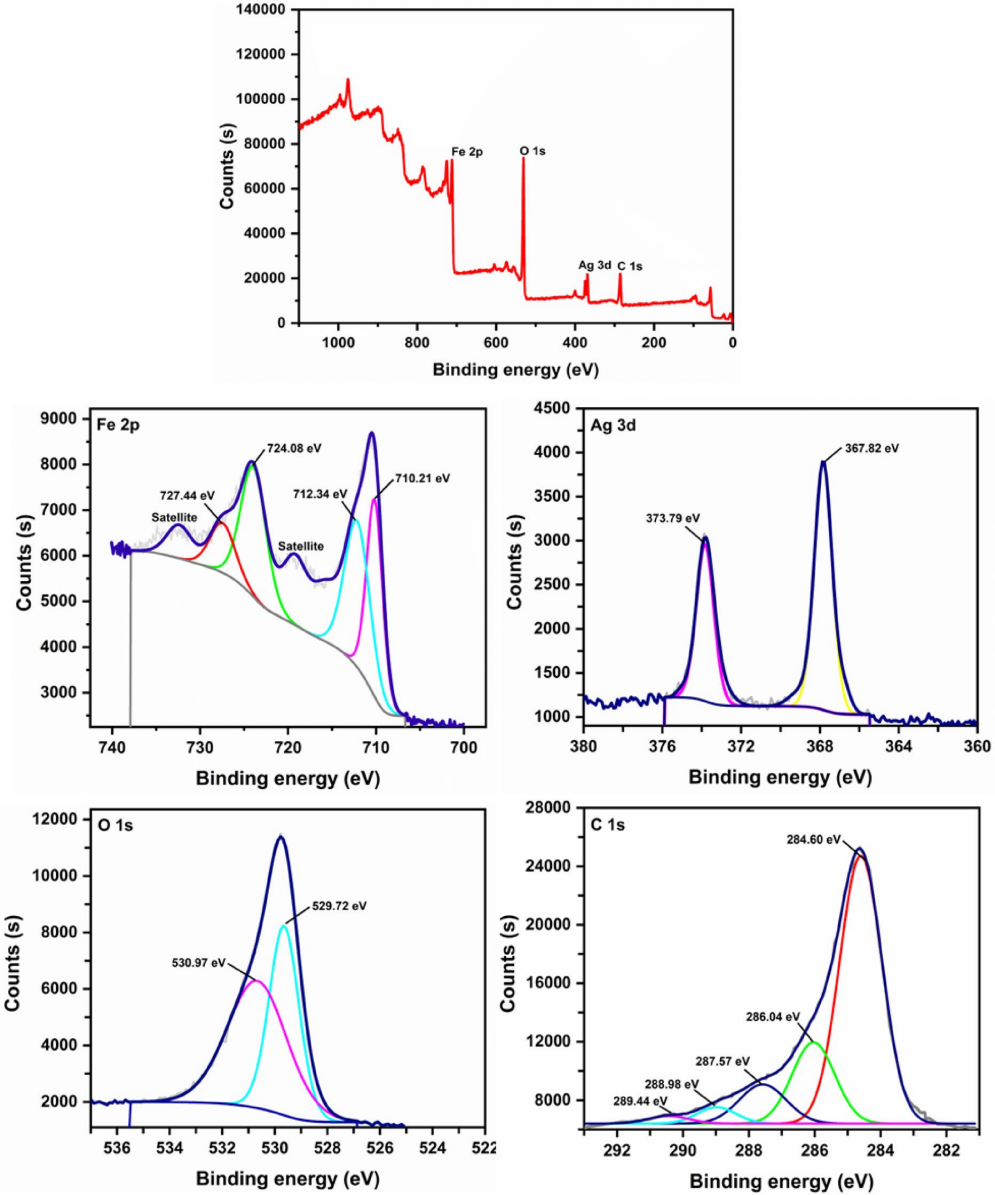
XPS survey spectrum of rGO/Fe_3_O_4_/Ag nanocomposite and high-resolution spectra of Fe 2p, Ag 3d, O 1s, and C 1s.

XPS analysis of the ternary nanocomposite (Fig. 3) confirmed the presence of Fe, O, Ag, and C elements. The high-resolution Fe 2p spectrum showing characteristic spin–orbit doublets at 710.42 eV (Fe 2p_3/2_) and 724.14 eV (Fe 2p_1/2_)^38,39^ along with deconvoluted peaks at 710.27 eV and 712.16 eV (Fe 2p_3/2_), and 724.08 eV and 727.15 eV (Fe 2p_1/2_) corresponding to Fe^2+^ and Fe^3+^ oxidation states, respectively^40,41^ while the satellite peaks at 719.25 eV and 732.46 eV^39^ further verified the formation of Fe_3_O_4_ NPs in the nanocomposite. The O 1s spectrum displayed two distinct components at 531.01 eV (surface hydroxyls, O–H) and 529.6 eV (lattice oxygen, Fe–O), verifying Fe_3_O_4_ incorporation in the nanocomposite^7,42,43^. The Ag 3d spectrum exhibited characteristic doublet peaks at 367.84 eV (3d_5/2_) and 373.82 eV (3d_3/2_) with a spin–orbit splitting of 6.0 eV, consistent with metallic silver (Ag⁰) formation^39,44^. The precise agreement of these binding energies with reference data confirms the complete reduction of silver ions to Ag⁰ nanoparticles in the nanocomposite^39,45^. The C 1s spectrum displays a dominant peak at 284.6 eV (sp^2^-hybridized carbon, C=C/C–C) along with small components at 286.0 eV (C–OH), 287.6 eV (C=O), and 288.9 eV (O–C=O)^46–48^. The relative intensity distribution, particularly the prominent sp^2^ carbon signal versus diminished oxygen-functionalized carbon peaks, confirms successful GO reduction to rGO in the ternary nanocomposite. XPS results confirm the successful incorporation of Fe, O, Ag, and C within the nanocomposite, along with their relevant oxidation states. The presence of metallic Ag⁰ and the coexistence of Fe^2+^/Fe^3+^ species suggest a strong potential for electron transfer processes. Predominant sp^2^-hybridized carbon domains and residual oxygen functionalities indicate partial restoration of the graphene’s π-conjugated system, alongside chemically active surface groups. These interfacial characteristics are expected to play a pivotal role in enhancing both adsorption capacity and catalytic efficiency. Additionally, the mixed-valence iron states confirm the formation of Fe_3_O_4_, which not only contributes to redox activity but also imparts magnetic properties, allowing for easy post-use separation.

#### Surface morphology and elemental analysis

**Fig. 4:**
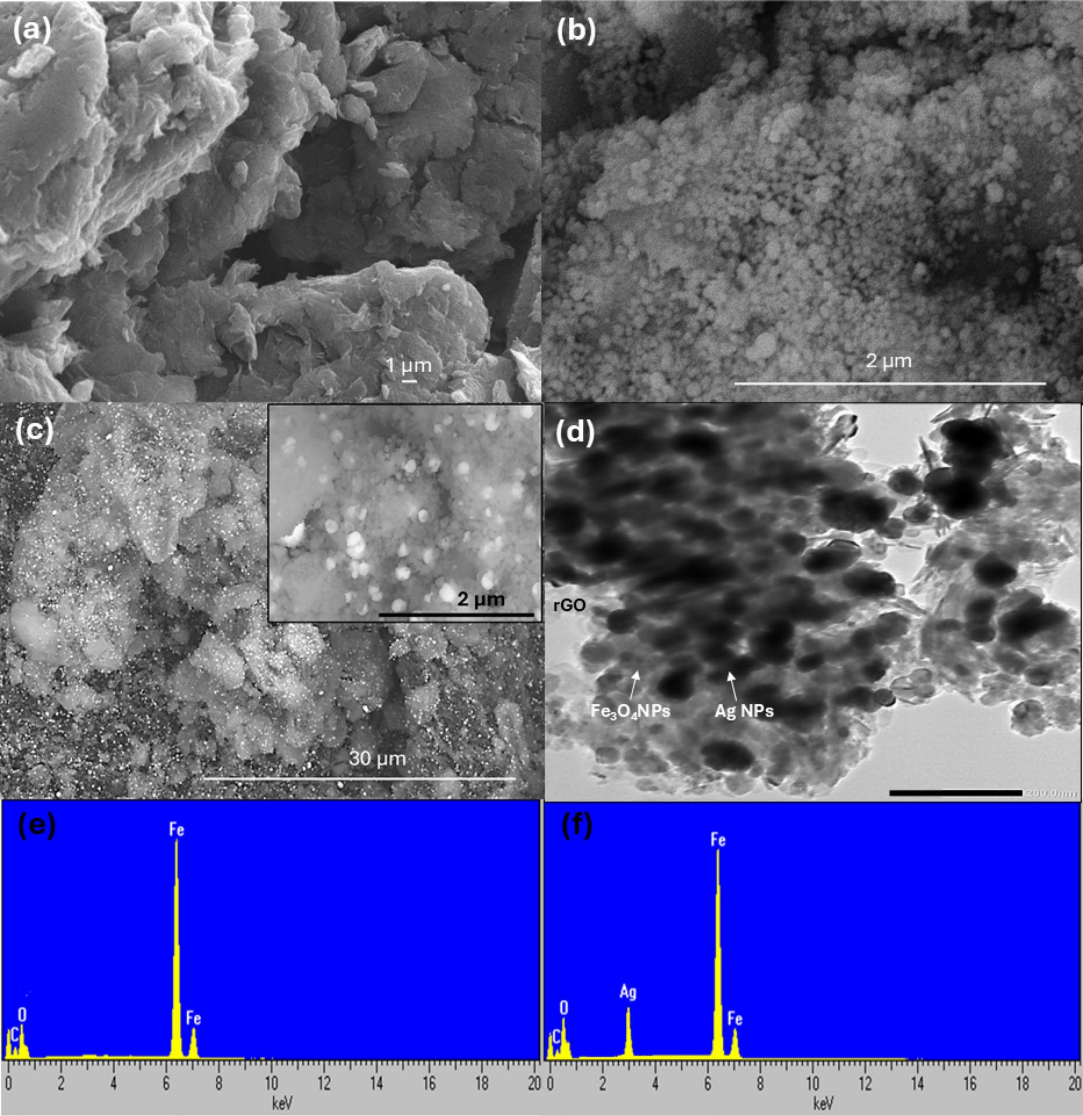
SEM images of (**a**) graphene oxide (GO), (**b**) the rGO/Fe_3_O_4_ binary nanocomposite, and (**c**) the rGO/Fe_3_O_4_/Ag ternary nanocomposite. (**d**) TEM image of the rGO/Fe_3_O_4_/Ag nanocomposite, along with EDX spectra of the nanocomposites shown in (**e**) and (**f**).

SEM distinctly reveals the morphological differences among GO, rGO/Fe_3_O_4_, and rGO/Fe_3_O_4_/Ag, emphasizing variations in surface topology and nanoparticles dispersion (Fig. 4). SEM image of GO typically reveal a layered, sheet-like structure with wrinkles and folds (Fig. 4a). For rGO/Fe_3_O_4_, the Fe_3_O_4_ NPs are often visible as uniformly distributed spherical spots or clusters on the rGO sheets, indicating good dispersion and interaction between the rGO and Fe_3_O_4_ NPs (Fig. 4b). For rGO/Fe_3_O_4_/Ag (Fig. 4c), the SEM image may show a similar morphology to rGO/Fe_3_O_4_ with additional features due to the presence of Ag NPs. These nanoparticles often appear as brighter spots on the rGO/Fe_3_O_4_ matrix, suggesting successful integration of Ag NPs. The EDX spectrum of rGO/Fe_3_O_4_ (Fig. 4e) exhibit characteristic emission lines for Fe (6.4–7.0 keV), O (0.52 keV), and C (0.28 keV), confirming the presence of iron oxide and rGO. In contrast, the rGO/Fe_3_O_4_/Ag spectrum (Fig. 4f) additionally shows a distinct Ag Lα peak at 2.98 keV, verifying the successful incorporation of Ag NPs. These findings align with XRD data, confirming successful synthesis and structural consistency. TEM analysis (Fig. 4d) was employed to resolve the nanoscale structural details and particle size distributions. The TEM micrograph reveals dense and uniform anchoring of Fe_3_O_4_ NPs on the rGO surface, with a narrow size distribution ranging from 6 to 18 nm. In contrast, Ag NPs appear darker and larger, with a broader size range of 8–45 nm. These findings confirm the successful anchoring of both nanoparticle types (Fe_3_O_4_ and Ag NPs) on the rGO support and further demonstrate the effectiveness of the synthesis approach.

### Adsorption studies

**Fig. 5:**
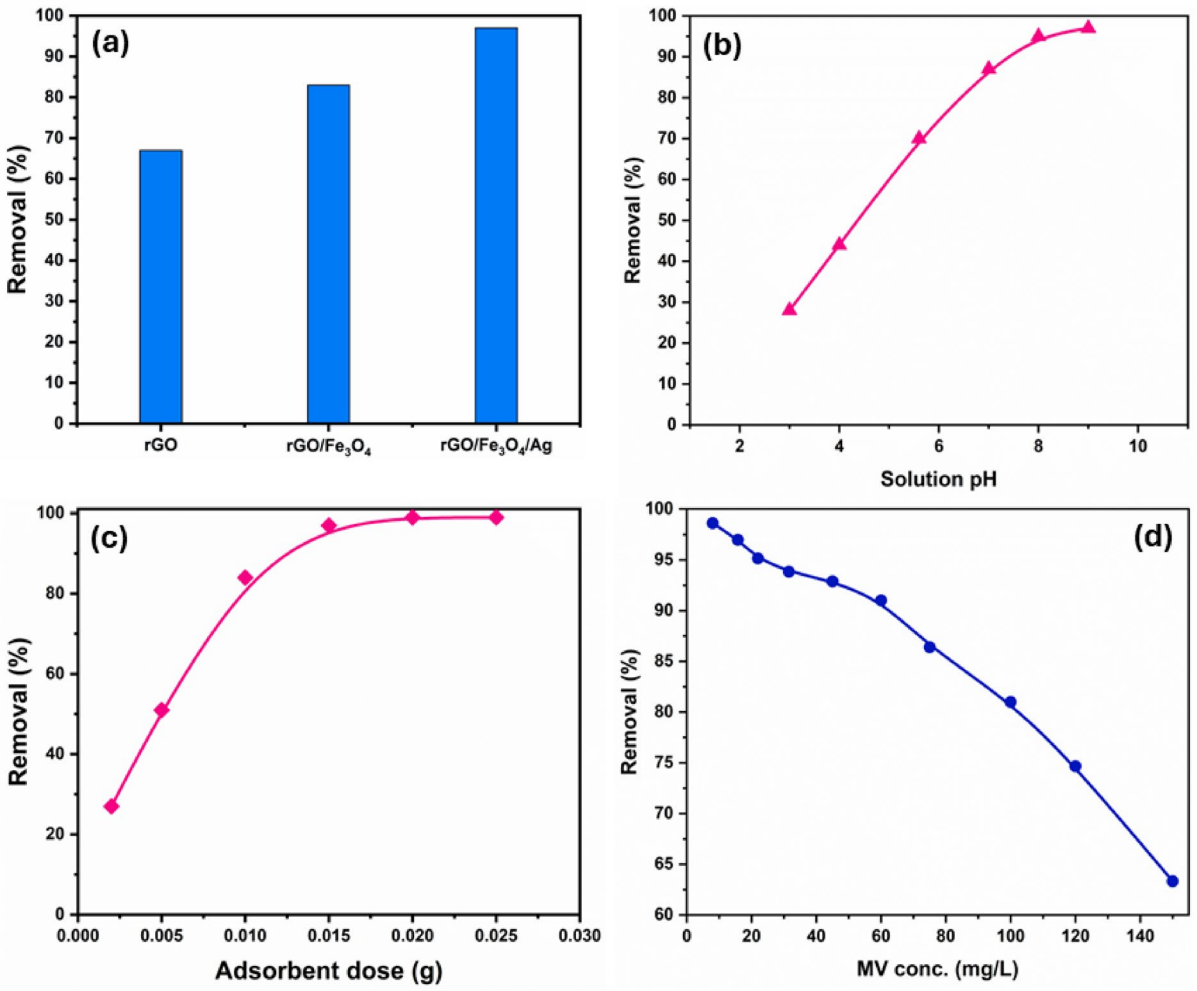
(**a**) Comparative MV removal efficiency of rGO, rGO/Fe_3_O_4_, and rGO/Fe_3_O_4_/Ag adsorbents (0.015 g). (**b**) Influence of pH on MV adsorption by rGO/Fe_3_O_4_/Ag (0.015 g, 30 °C). (**c**) Effect of rGO/F_3_O_4_/Ag dosage on MV removal; and (**d**) removal efficiency at varying MV concentrations using 0.015 g rGO/F_3_O_4_/Ag at pH 9 and 30 °C.

Nanocomposites usually exceed their individual components in terms of dye adsorption due to their synergistic effects, increased surface area, stability, selectivity, and regeneration capabilities. As a result, the adsorption capability of the ternary nanocomposite (rGO/Fe_3_O_4_/Ag) was compared to rGO and rGO/Fe_3_O_4_ nanocomposite in the removal of MV dye. To assess the effectiveness of their elimination, the following parameters were used: [MV]_0_ = 15.75 mg/L, dosage = 0.015 g, pH = 9 at 30 °C, and rotation speed = 130 rpm. Figure 5a indicates that the rGO/Fe_3_O_4_/Ag nanocomposite exhibits the highest adsorption efficiency (97%). This enhanced performance can be attributed to the synergistic interactions among its constituent components, which significantly enhances its overall performance in the MV adsorption process. This was supported by an increased surface area, pore volume, and pore size of the ternary nanocomposite, as confirmed by BET and BJH analysis.

#### Factors affecting MV removal efficiency

While performing an adsorption process to target a specific pollutant, it is vital to study the variable parameters that exert effect. These include pollutant concentration, aqueous solution pH, adsorbent dose, and temperature. Optimization of these conditions will be critical to the advancement of large-scale pollutant removal technology. This study explored the impact of these parameters on MV dye adsorption using the ternary nanocomposite.

##### pH effect

The dye solution’s pH significantly influences both adsorption efficiency and equilibrium uptake capacity. pH values can alter the ionization of certain pollutants as well as the charge of absorbent material. At pH levels ranging from 3 to 9, the adsorption efficacy of rGO/Fe_3_O_4_/Ag (0.015 g) for MV (15.75 mg/L) was evaluated. Figure 5b illustrates that as pH increases from 3 to 9, MV removal efficiency improves from 28 to 97%. Under acidic conditions, protonation generates positive surface charges that electrostatically repel cationic dye molecules, limiting adsorption. With increasing pH, the adsorbent develops a negative surface charge that improves dye adsorption through stronger electrostatic interactions. Therefore, the optimal pH for MV dye adsorption using rGO/Fe_3_O_4_/Ag is 9.

##### Nanocomposite dose

The uptake efficiency of a specific adsorbate by a solid adsorbent is primarily determined by the amount of adsorbent used. Figure 5c illustrates the effect of varying doses of rGO/Fe_3_O_4_/Ag nanocomposite on MV adsorption. Raising the nanocomposite amount from 0.002 g to 0.015 g elevated MV adsorption efficiency from 27 to 97%, primarily due to the amplified presence of active sites enabling more effective dye binding. At higher dosages (0.020–0.025 g), MV removal efficiency plateaued at ~ 99%, suggesting near-complete occupation of adsorption sites and limited additional uptake.

##### MV initial concentration

Adsorption behavior is strongly influenced by the initial adsorbate concentration, as it dictates the maximum uptake achievable by a given quantity of adsorbent. MV adsorption by rGO/Fe_3_O_4_/Ag nanocomposite was evaluated across a concentration range of 8 to 150 mg/L using 0.015 g of the nanocomposite. The results showed a decline in removal efficiency as the initial MV concentration increased (Fig. 5d). At higher dye concentrations, the number of dye molecules increases, but due to the limited number of adsorption sites, the adsorbent becomes saturated more quickly, resulting in a lower percentage of dye removal.

#### Adsorption kinetics

MV adsorption showed rapid initial uptake, reaching ~ 85% within 20 min due to abundant active sites. The rate then slowed as sites became occupied, approaching equilibrium by 60 min, with a final removal efficiency of 97%. The adsorption kinetics of MV on the rGO/Fe_3_O_4_/Ag surface are interpreted via pseudo-1^st^-order^49^, pseudo-2^nd^-order^50^, and intraparticle diffusion^51^ models, which clarify the adsorption rate and underlying mechanism. Table 1 contains the non-linear equations for these models as well as the calculated values of their parameters. *qe* and qt denote MV adsorption capacity at equilibrium and at time t, respectively, expressed in mg/g. The adsorption kinetics were represented by three rate constants: k_1_ (pseudo-1^st^-order, min^−1^), k_2_ (pseudo-2^nd^-order, g/mg min), and k_p_ (intraparticle diffusion, mg/g min^0.5^). The boundary layer thickness is denoted by C (mg/g). To evaluate each model’s fit with experimental data, three error functions—sum of squares error (SSE), root mean square error (RMSE), and reduced chi-square (Reduced χ^2^)—were employed alongside correlation coefficient values (R^2^). The functions are represented by the following equations (Eqs. 5–7)^52^.

5 $$\text{SSE}=\sum_{\text{i}=1}^{\text{n}}({\text{q}}_{\text{exp}}-{\text{ q}}_{\text{cal}}{)}^{2}$$

6 $$\text{RMSE}=\sqrt{\frac{1}{\text{n}-2 }\sum_{\text{i}=1}^{\text{n}}({\text{q}}_{\text{exp}- }{\text{q}}_{\text{cal}}}{)}^{2}$$

7 $$Reduced\;\chi^{2} = \frac{{\mathop \sum \nolimits_{i = 1}^{n} \frac{{\left( {q_{\exp } - q_{cal} } \right)^{2} }}{{q_{cal} }}}}{v}$$

**Table 1:** Kinetic parameters for MV dye adsorption onto rGO/Fe_3_O_4_/Ag nanocomposite (0.015 g) at pH 9 and 30 °C.

Kinetic models	Equations	Parameters	Values
pseudo-1^st^-order	${q}_{t}={q}_{e}\left(1-{e}^{-{k}_{1}t}\right)$	q_e exp_ (mg/g)	25.62 ± 0.40
		q_e cal_ (mg/g)	24.33 ± 0.63
		k_1_ (min^−1^)	0.1886 ± 0.03
		R^2^	0.8328
		SSE	38.259
		RMSE	1.864
		Reduced χ^2^	3.478
pseudo-2^nd^-order	${q}_{t}=\frac{{k}_{2} {q}_{e}^{2} t}{1 + {k}_{2 }{q}_{\text{e }}t}$	q_e exp_ (mg/g)	25.62 ± 0.40
		q_e cal_ (mg/g)	26.57 ± 0.49
		k_2_ (g/mg min)	0.011 ± 0.001
		R^2^	0.9551
		SSE	10.269
		RMSE	0.966
		Reduced χ^2^	0.933
Intraparticle diffusion	${q}_{t}={k}_{p}{ t}^{0.5}+C$	k_p1_ (mg/g min^0.5^)	3.233 ± 0.10
		C (mg/g)	7.778 ± 0.36
		R^2^	0.9980
		SSE	1.290
		RMSE	0.651
		Reduced χ^2^	0.430
		k_p2_ (mg/g min^0.5^)	0.448 ± 0.04
		C (mg/g)	21.868 ± 0.28
		R^2^	0.9810
		SSE	0.063
		RMSE	0.112
		Reduced χ^2^	0.012

**Fig. 6:**
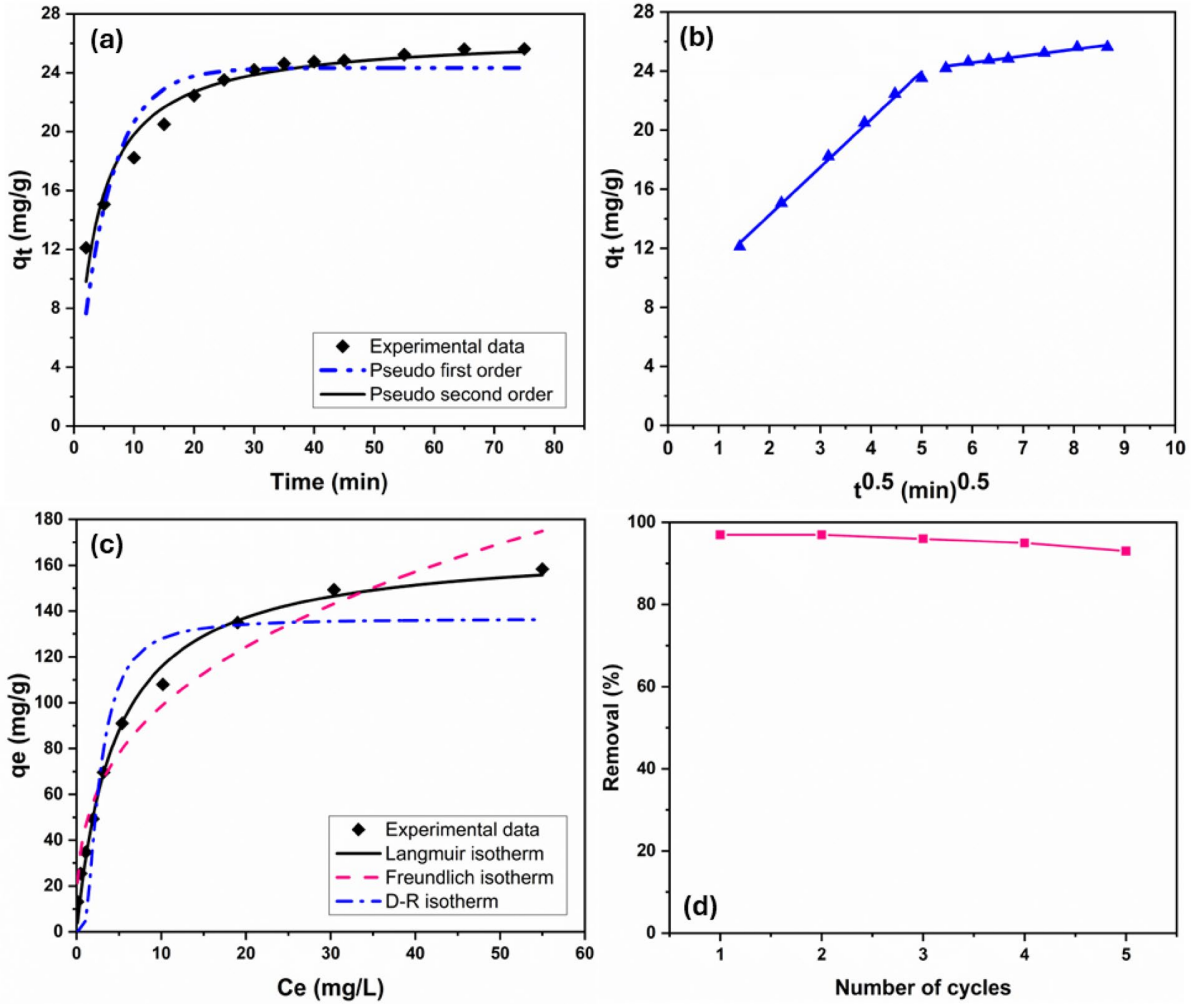
(**a**) Non-linear plots for pseudo-1^st^ and 2^nd^ order kinetics; (**b**) Linearized intraparticle diffusion model for MV adsorption (conditions: pH 9, 30 °C); (**c**) Non-linear regression fits of Langmuir, Freundlich, and D-R isotherm models; (**d**) Reusability assessment of rGO/Fe_3_O_4_/Ag nanocomposite (0.015 g) over multiple MV adsorption cycles.

The variables q_cal_ and q_exp_ correspond to calculated and experimental data, respectively, with n denoting the number of observations and $v$ representing the degrees of freedom. Data fitting and error analysis were performed using OriginPro. The best-fit models showed the highest R^2^ and lowest errors. Figure 6a displays non-linear fitting curves for kinetic models (pseudo-1^st^ and pseudo-2^nd^-order). The pseudo-1^st^-order model, which relates the adsorption rate to the number of vacant sites^53^, gave a calculated equilibrium capacity (q_e_,cal) of 24.33 mg/g, slightly below the experimental value (q_e_,exp = 25.62 mg/g), as listed in Table 1. Its moderate correlation (R^2^ = 0.8328) and higher error indices (SSE = 38.259, RMSE = 1.864, reduced χ^2^ = 3.478) suggest a weaker fit to the experimental data. In contrast, the pseudo-2^nd^-order model showed better agreement, with q_e_,cal = 26.57 mg/g and superior statistical outcomes (R^2^ = 0.9551, SSE = 10.269, RMSE = 0.966, reduced χ^2^ = 0.933), also detailed in Table 1. This model presumes that the rate depends on the square of available active sites, indicating that adsorption may involve more complex surface interactions^53,54^. Nevertheless, as an empirical model, it does not confirm the exact mechanism (e.g., physical vs. chemical adsorption), but rather implies that uptake kinetics are more governed by surface site availability than by dye concentration in solution.

Dye adsorption typically involves two main transport steps: external mass transfer (EMT) across the boundary layer and intraparticle diffusion (IPD) within the adsorbent’s pores^52^. To elucidate the diffusion dynamics, the IPD model was employed. The resulting plot displayed two linear segments (Fig. 6b), indicating a multi-step process. The initial phase, marked by a higher diffusion rate (kp_1_ = 3.233 mg/g min^0.5^^5^), is attributed to rapid EMT. The subsequent phase, with a lower rate constant (kp_2_ = 0.448 mg/g min^0.5^^5^), reflects slower IPD through internal pores. Since the plot did not intersect the origin (Fig. 6b), IPD alone does not govern the overall rate, suggesting that boundary layer effects are also significant^52^. The intercepts (C = 7.778 and 21.868 mg/g) further support the contribution of surface diffusion in the initial adsorption stage.

#### Adsorption thermodynamics

Adsorption performance is strongly influenced by temperature. MV uptake was measured at 25, 30, and 35 °C using 0.015 g of rGO/Fe_3_O_4_/Ag nanocomposite. MV elimination effectiveness rose from 85.1 to 99.7% with temperature increase (25–35 °C), indicating an endothermic adsorption behavior. The thermodynamic parameters—ΔG° (standard Gibbs free energy change), ΔS° (standard entropy change), and ΔH° (standard enthalpy change)—were computed using Eqs. (8) and (9). The distribution constant (K_d_) was derived from the equilibrium adsorption capacity (q_e_, mg/g) and the residual concentration (C_e_, mg/L) in the liquid phase. All calculations were performed with temperature (T) expressed in Kelvin, and the universal gas constant (R = 8.314 J/mol K) was applied.

8 $$\text{ln }{\text{k}}_{d} =\frac{{\Delta S}^{o}}{R}-\frac{{\Delta H}^{o}}{RT}$$

9 $$\Delta {G}^{o}= {\Delta H}^{o}-T{\Delta S}^{o}$$

The ΔH° and ΔS° values were derived from the van’t Hoff plot of ln K_d_ against inverse temperature (1/T) (Fig. 8). Table 2 reveals consistently negative ΔG° across all studied temperature ranges, demonstrating the spontaneous nature and thermodynamic feasibility of MV adsorption onto the rGO/Fe_3_O_4_/Ag nanocomposite^55^. As the temperature rises, the negative values of ΔG° increase, implying that the nanocomposite’s efficiency in eliminating MV is significantly boosted^56^. The positive ΔH° (32.42 kJ/mol) confirms that MV adsorption on nanocomposite is endothermic. Since this value is below 40 kJ/mol, the process aligns with physisorption. The positive ΔS° reflects increased randomness at the solid–liquid boundary, supporting electrostatic-driven physical adsorption.

**Table 2:** Thermodynamic parameters for MV adsorption on rGO/Fe_3_O_4_/Ag at various temperatures.

Temperature (°C)	ΔG° (kJ/mol)	ΔH° (kJ/mol)	ΔS° (J/mol K)
25	− 09.449		
30	− 10.152	32.42	140.50
35	− 10.854		

#### Adsorption isotherm analysis

Isotherm models are widely applied to analyze equilibrium interactions between adsorbent and adsorbate, revealing critical data on the adsorption capacity limit. MV adsorption on rGO/Fe_3_O_4_/Ag nanocomposite was evaluated using three models: Freundlich^57^ (surface heterogeneity), Dubinin-Radushkevich (D-R)^58^ (adsorption energy, E), and Langmuir (monolayer capacity)^59^. Table 3 lists their non-linear equations, model parameters, error values, and R^2^. In Freundlich, n denotes adsorption intensity, and k_F_ (mg/g)(L/mg)^1/n^ indicates adsorption capacity. For Langmuir, k_L_ (L/mg) represents the isotherm constant, and q_max_ (mg/g) denotes the monolayer adsorption limit. The D–R model distinguishes physical from chemical adsorption by calculating E (kJ/mol) based on Polanyi potential (ε, kJ/mol) and slope constant β (mol^2^/kJ^2^), while theoretical capacity q_s_ (mg/g) is also determined. Figure 6c presents the non-linear isotherm fits for Freundlich, Langmuir, and D–R models.

**Table 3:** Isotherm parameters for MV adsorption onto rGO/Fe_3_O_4_/Ag nanocomposite; experimental conditions (adsorbent dose = 0.015 g, pH = 9, T = 30 °C).

Isotherm models	Equations	Parameters	Values
Langmuir	${\text{q}}_{\text{e}}=\frac{{\text{q}}_{\text{max}}{\text{C}}_{\text{e}} {\text{K}}_{\text{L}}}{1 +{\text{ C}}_{\text{e}}{\text{K}}_{\text{L}}}$	q_max_ (mg/g)	168.70 ± 5.27
		k_L_ (L/mg)	0.2177 ± 0.02
		R^2^	0.9892
		SSE	272.88
		RMSE	05.840
		Reduced χ^2^	34.109
Freundlich	${\text{q}}_{\text{e}}={\text{k}}_{\text{F}} {{\text{C}}_{\text{e}}}^{\frac{1}{\text{n}}}$	n	02.96 ± 0.03
		k_F_ ((mg/g)(L/mg)^1/n^)	45.277 ± 4.50
		R^2^	0.9539
		SSE	1034.62
		RMSE	11.372
		Reduced χ^2^	129.33
Dubinin-Radushkevich	${\text{q}}_{\text{e}}={\text{q}}_{\text{s}}{\text{ e}}^{-\upbeta {\upvarepsilon }^{2}}$ $\upvarepsilon =\text{RTln}\left(\frac{1}{{\text{C}}_{\text{e}}}+1\right)$ $\text{E}=\frac{1}{\sqrt{2\upbeta }}$	q_s_ (mg/g)	136.60 ± 10.30
		β (mol^2^ k/J)	1.168 ± 0.37
		E (kJ/mol)	0.654
		R^2^	0.8673
		SSE	3354.64
		RMSE	20.477
		Reduced χ^2^	419.33

According to Table 3, the Langmuir model showed the best agreement with experimental data (R^2^ = 0.9892, SSE = 272.88, RMSE = 5.840, χ^2^ = 34.109), indicating monolayer adsorption on a homogeneous surface^10^. The calculated maximum capacity (q_max_) was 168.70 ± 5.27 mg/g, reflecting strong affinity between MV dye and the rGO/Fe_3_O_4_/Ag nanocomposite. The Freundlich model, which assumes surface heterogeneity and multilayer adsorption^19^, gave a slightly lower correlation (R^2^ = 0.9539) and higher error values. The adsorption intensity (n = 2.96) suggests favorable uptake, as n > 1 typically reflects strong adsorbent–adsorbate interaction. The Dubinin–Radushkevich model, despite its weaker fit (R^2^ = 0.8673, highest errors), provides insight into the mechanism. The calculated mean free energy (E = 0.654 kJ/mol) is well below 8 kJ/mol, suggesting that the process is predominantly physisorption^19^, likely governed by weak van der Waals forces rather than chemical bonding^52^.

#### Adsorbent reuse

The recycling of adsorbents is vital for sustainable wastewater treatment, as it extends their lifespan, ensures efficient pollutant removal, and supports environmentally friendly and cost-effective processes. This study evaluated the reusability of rGO/Fe_3_O_4_/Ag nanocomposites for removing methyl violet (MV) from aqueous solutions. MV desorption was accomplished by immersing the nanocomposite in a 0.1 N HCl solution, followed by rinsing with 10% ethanol. Due to the superparamagnetic properties of the adsorbent, it was easily recovered from the mixture using a magnet, allowing for reuse in successive adsorption cycles (Fig. 6d). The rGO/Fe_3_O_4_/Ag nanocomposite maintained high MV removal efficiency across five cycles under 0.015 g dosage, 15.75 mg/L initial concentration, and 30 °C, with only ~ 4.3% efficiency loss. This combination of recyclability and stable performance positions rGO/Fe_3_O_4_/Ag as a sustainable alternative for dye pollution mitigation in water treatment applications.

#### Proposed mechanism for MV dye adsorption

**Scheme 2:**
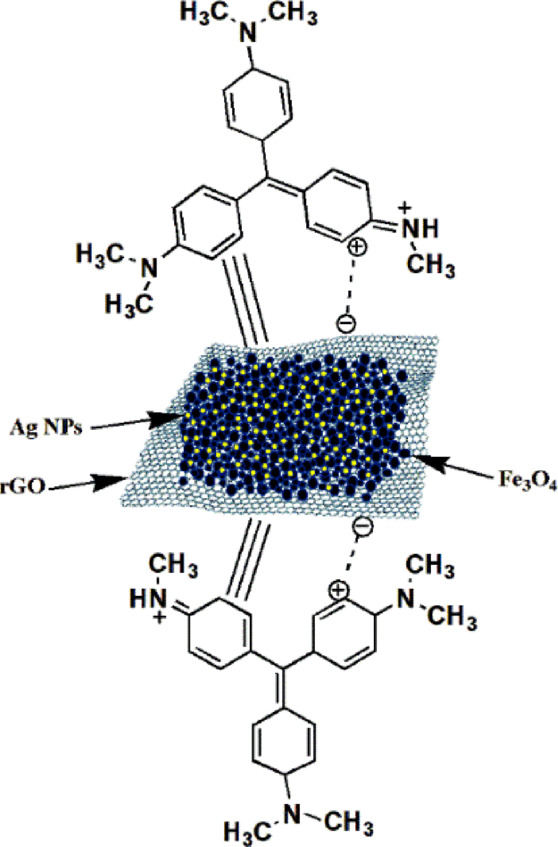
Proposed adsorption mechanism of MV on rGO/Fe_3_O_4_/Ag nanocomposite.

The adsorption of MV dye onto the rGO/Fe_3_O_4_/Ag nanocomposite is primarily governed by electrostatic interactions and π–π stacking, as illustrated in the proposed mechanism (Scheme 2). Under neutral to alkaline conditions, MV predominantly exists in its cationic form (pKₐ ≈ 9.17). At these pH levels, the deprotonation of surface hydroxyl groups on the nanocomposite increases its negative surface charge, thereby enhancing the electrostatic interaction between the nanocomposite and the positively charged MV molecules. Simultaneously, π–π stacking between MV’s aromatic rings and rGO’s conjugated graphitic domains promotes additional non-covalent adsorption. Furthermore, the incorporation of both Ag and Fe_3_O_4_ NPs introduces additional active sites. This synergistic combination markedly elevates the nanocomposite’s adsorption capacity and dye removal efficiency. To elucidate the adsorption mechanism of MV onto the rGO/Fe_3_O_4_/Ag nanocomposite, FTIR spectra of both pure MV and the nanocomposite after adsorption were analyzed (Fig. 1b). The MV spectrum exhibited multiple characteristic bands at 2916–2867 cm^−1^ (aliphatic C–H stretching), 1592 cm^−1^ (aromatic C=C stretching), 1462 cm^−1^ (–CH_3_ bending), 1380 cm^−1^ (C–N stretching), and 1178 cm^−1^ (aromatic C–H bending)^60^. These peaks persisted in the MV-loaded nanocomposite, albeit with reduced intensity and slight shifts, indicating effective adsorption without structural degradation. The observed spectral changes suggest that adsorption is primarily driven by π–π stacking between MV’s aromatic rings and the rGO domains, as well as electrostatic interactions between the negatively charged surface and cationic dye molecules. Furthermore, the decreased intensity of the O–H stretching band at 3441 cm^−1^ after adsorption suggests hydrogen bonding interactions between MV molecules and surface hydroxyl groups.

XPS analysis supports the proposed adsorption mechanism by confirming key surface features of the nanocomposite. The presence of sp^2^-hybridized carbon enables π–π interactions with MV’s aromatic rings, while oxygen-containing groups (e.g., C–O, O–C=O) facilitate electrostatic binding with the cationic dye. The coexistence of Fe^2^⁺ and Fe^3^⁺ confirms the formation of Fe_3_O_4_, contributing to the material’s magnetic separability. Additionally, Ag NPs enhance surface conductivity, act as adsorption sites for MV, and serve as the main catalytic centers for the reduction of *p*-NA, emphasizing their multifunctional role.

#### Assessment of rGO/Fe_3_O_4_/Ag’s adsorption capacity

The rGO/Fe_3_O_4_/Ag nanocomposite outperforms many previously reported adsorbents in MV removal capacity (Table 4). Its magnetic nature also enables quick and efficient separation via an external magnetic field, making it a strong candidate for wastewater treatment applications.

**Table 4:** Assessment of adsorption capacities for MV dye in simulated wastewater.

Adsorbents	q_max_ (mg/g)	References
HNT–Fe₃O₄	20.04	61
algal activated carbon/Fe₃O₄ magnetic composite	59.88	62
Alginate-coated perlite beads	4.98	63
CMC/g-C₃N₄/ZnO	96.43	64
kaolin/CuFe₂O₄ composite	117.65	65
MMT/GO/CoFe₂O₄	97.26	66
β-cyclodextrin immobilised onto mesoporous silica	36.25	67
ACOW600/ZnO/Fe₃O₄	48.59	68
Magnetic chitosan microspheres	128.84	69
Clay/starch/Fe₃O₄ nanocomposite	29.67	70
KCZF (Sono-assisted adsorption)	08.18	71
CS-PVA/FGA/MMT	105.7	72
KAlPO₄F	29.41	73
Magnetic kaolin/TiO₂/γ-Fe₂O₃ nanocomposite	131.58	74
rGO/Fe₃O₄/Ag nanocomposite	168.70	This work

### Kinetic studies of *p*-nitroaniline (*p*-NA) reduction

**Fig. 7:**
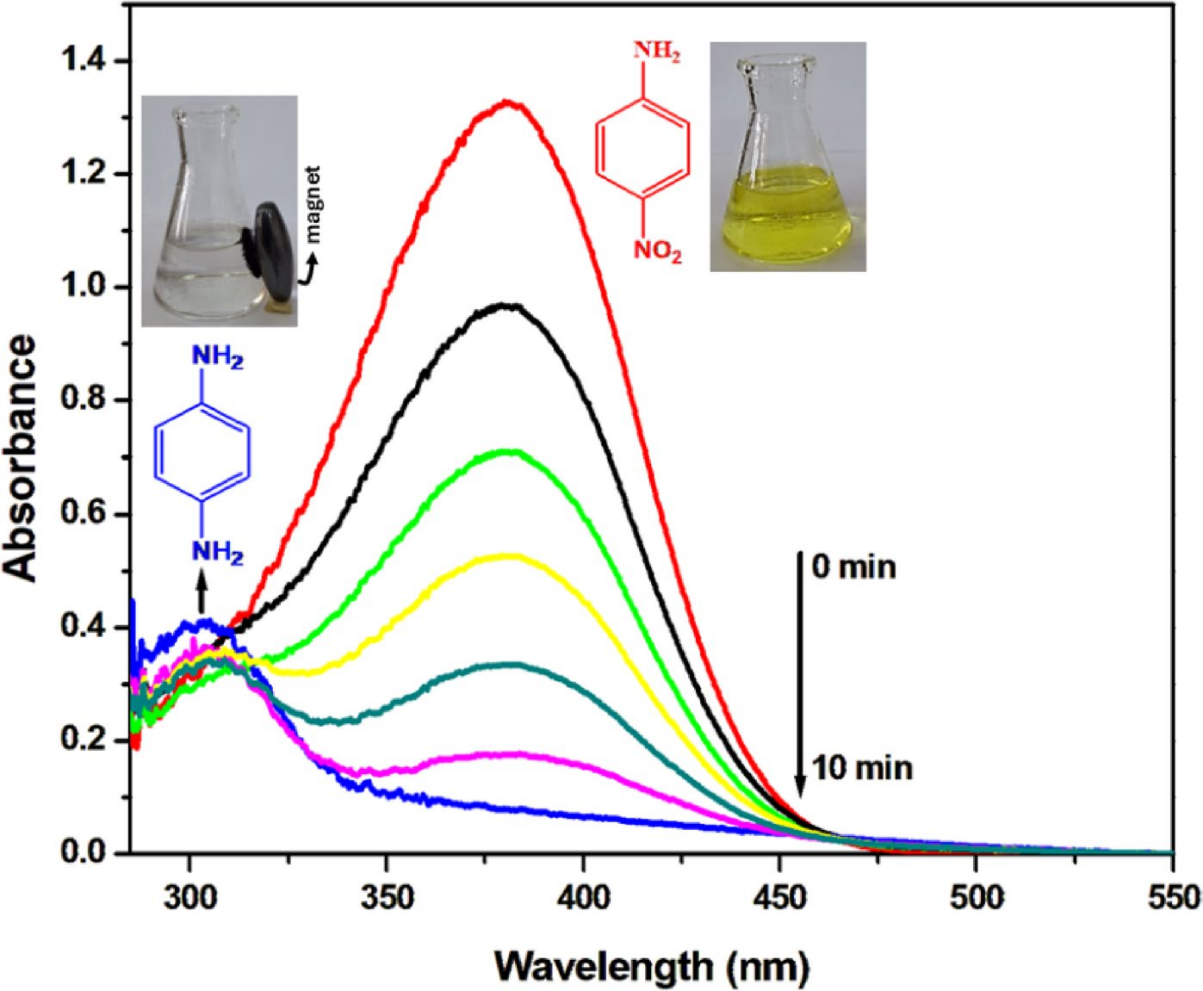
Absorption spectra of *p*-NA (0.1 mM) measured at different time intervals during its reduction using 0.005 g of catalyst and 8 mM NaBH_4_ at 27 °C. The inset images show the decolorization of *p*-NA (yellow) to *p*-PDA (colorless) after reduction.

Catalytic conversion of para-nitroaniline (*p*-NA, C_6_H_6_N_2_O_2_) to para-phenylenediamine (*p*-PDA, C_6_H_8_N_2_) holds industrial significance, as *p*-PDA serves as a key intermediate in manufacturing polymers, hair dyes, and rubber products. Spectroscopic analysis of the *p*-NA/NaBH_4_ reduction system demonstrated profound kinetic limitations in the absence of catalyst. The uncatalyzed reaction showed minimal progress, with only 2.1 ± 0.3% in the nitro group’s absorbance at 380 nm, with no detectable formation of the 307 nm band corresponding to *p*-PDA, indicating negligible conversion. Upon introduction of the rGO/Fe_3_O_4_/Ag nanocomposite, the reaction proceeded rapidly, reflected by the sharp decline of the 380 nm band and the concurrent formation of the 307 nm band (Fig. 7). These spectral shifts confirm efficient electron transfer from BH_4_^−^ to *p*-NA and nearly complete transformation into *p*-PDA ^75,76^. The deep yellow solution of *p*-NA turned colorless, visually indicating the formation of *p*-PDA (inset of Fig. 7). The rGO/Fe_3_O_4_/Ag nanocomposite exhibited exceptional catalytic proficiency, achieving 95.7 ± 0.5% conversion within 10 min. This performance markedly surpasses that of the rGO/Fe_3_O_4_ nanocomposite, which reached only 15.2 ± 0.2% conversion after 24 h. The enhanced catalytic activity is attributed to the incorporation of Ag NPs, which introduce abundant active sites and promote rapid electron transfer, thereby reducing kinetic barriers and accelerating reduction rate. This synergistic ternary design underscores the critical role of Ag in optimizing catalytic functionality. Notably, analogous performance enhancements have been observed in the Fe_3_O_4_/SiO_2_/Ag nanocomposite system^13^. Reaction kinetics were evaluated using a pseudo-first-order treatment due to the substantial molar excess of NaBH_4_ over *p*-NA. The relationship followed the equation ln (A₀/A_t_) = k_o_t, where A₀ and A_t_ represent the absorbance of *p*-NA at λ_max_ = 380 nm initially and at time t, respectively, and k_o_ denotes the observed rate constant (min^−1^). The catalytic efficiency is in line with XPS findings, which confirmed the presence of metallic Ag⁰ NPs—crucial for enhancing electron transfer—and Fe^2+^/Fe^3+^ redox couples that facilitate the reduction process. The presence of Fe_3_O_4_ imparts magnetic properties, allowing rapid catalyst recovery and reuse without loss in activity—an essential feature for practical wastewater treatment applications.

#### Effect of NaBH_4_ concentration

**Fig. 8:**
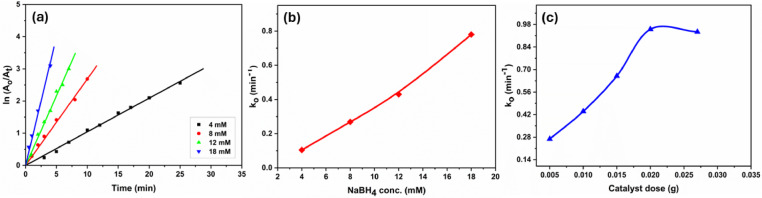
(**a**) Pseudo-first-order kinetic plots and (**b**) corresponding rate constants for the catalytic reduction of *p*-NA (0.1 mM) using 0.005 g of rGO/Fe_3_O_4_/Ag catalyst at 27 °C under varying NaBH_4_ concentrations; (**c**) Variation of rate constants for *p*-NA reduction (27 °C, 8 mM NaBH_4_) at different catalyst doses.

The concentration of NaBH_4_ plays a crucial role in driving the reduction of *p*-NA by supplying electrons through borohydride ions (BH_4_^−^), which act as both electron donors and hydrogen sources. The reduction rate was examined across NaBH_4_ concentrations ranging from 4 to 18 mM. A strong linear correlation (R^2^ > 0.99) between ln (A₀/A_t_) and reaction time (t) confirmed pseudo-first-order kinetics (Fig. 8a). The corresponding rate constant (k_o_) increased linearly with NaBH_4_ concentration, reaching a maximum of 0.78 min^−1^ at 18 mM (Fig. 8b). Upon adsorption of both *p*-NA and BH_4_^−^ onto the rGO/Fe_3_O_4_/Ag nanocomposite surface, BH_4_^−^ underwent hydrolysis to release active hydrogen species while concurrently transferring electrons to *p*-NA, facilitating rapid hydrogenation of the nitro group. This dual mechanism enabled complete conversion within 4 min, underscoring both the catalytic efficiency and the critical role of BH_4_^−^ concentration in governing the reaction kinetics.

#### Effect of catalyst dose

The catalytic efficiency of rGO/Fe_3_O_4_/Ag is significantly influenced by its dosage. To evaluate the impact of catalyst dosage on *p*-NA reduction efficiency, reactions were conducted using different amounts of rGO/Fe_3_O_4_/Ag while maintaining constant concentrations of NaBH_4_ (8 mM) and *p*-NA (0.1 mM) at 27 °C. As illustrated in Fig. 8c, the reduction rate increased with catalyst dosage, going from 0.005 g to 0.015 g. This enhancement is most likely due to a higher number of active sites on the rGO/Fe_3_O_4_/Ag catalyst surface, which boosts reaction efficiency. At an optimal dose of 0.02 g, the reduction occurs in just 3 min, reaching a maximum k_o_ value of 0.95 min^−1^. However, beyond this dose, the improvement plateaus due to nanoparticle aggregation, which reduces the availability of active sites.

#### Comparative activity and reusability of the catalyst

The rGO/Fe_3_O_4_/Ag catalyst can be easily recovered from the reaction mixture via magnetic separation (Fig. 7), enabling efficient reuse. Following each cycle, the catalyst was rinsed with distilled H_2_O, dried thoroughly, and reused under identical conditions (0.1 mM *p*-NA, 8 mM NaBH_4_, 0.02 g catalyst, 27 °C). UV–vis analysis revealed consistent catalytic performance over eight consecutive cycles, with no significant variation in reduction time and a maintained conversion efficiency of ~ 97.6%. These results confirm the catalyst’s robust stability and excellent reusability. In addition, ICP-OES (inductively coupled plasma–optical emission spectrometry) analysis showed no detectable leaching of Fe or Ag ions into the solution, verifying the chemical stability of the nanocomposite during repeated use. Compared to previously reported catalysts (Table 5), the rGO/Fe_3_O_4_/Ag nanocomposite demonstrated exceptional catalytic activity in the reduction of *p*-NA, exhibiting marked improvements over earlier systems. These results highlight the synergistic effect of the composite’s ternary structure, which combines high catalytic efficiency with excellent magnetic recoverability and chemical stability.

**Table 5:** Rate constants and reaction times for the reduction of *p*-NA using various catalytic systems.

Catalyst	k_o_ (min^−1^)	Reduction time (min)	References
Ag NWs–rGO	0.6132	4	^77^
Pt NPs/GA composite	0.44	10	^78^
AgP(NIPAM-AAc-AAm) hybrid microgels	0.606	–	^79^
ZnO/CdO/RGO	7.1 × 10^−3^	–	^80^
RGO-Ni	1.296 × 10^−2^	190	^81^
Ag–p(NiPA-co-AAc) hybrid microgels	0.4148	13	^82^
Cobalt (II) complex	0.092	15	^83^
SiO_2_/NiFe_2_O_4_ NC	3.1 × 10^−1^	30	^84^
CeO_2_/Bi_2_WO_6_	0.38	4	^85^
rGO/Fe_3_O_4_/Ag	0.95	3	This work

## Conclusion

The ternary rGO/Fe_3_O_4_/Ag nanocomposite was successfully synthesized via a one step hydrothermal method. Characterization results confirmed its well-defined structure, superparamagnetic nature, favorable surface and pore characteristics. The nanocomposite exhibited exceptional adsorption capacity for methyl violet 2B (MV) dye from aqueous solutions, with performance optimized by varying adsorbent dosage, pH, initial dye concentration, and temperature. The rGO/Fe_3_O_4_/Ag nanocomposite displayed remarkable adsorption capacity (q_max_ = 168.70 mg/g), attributed to synergistic effects among its constituents. The adsorption behavior conformed to pseudo-2^nd^-order kinetics and fitted Langmuir model, indicating monolayer surface coverage. Apart from its adsorption capability, the nanocomposite served as a highly efficient catalyst in the NaBH_4_-assisted reduction of *p*-NA to *p*-PDA, achieving 97.6% conversion within 3 min and a rate constant (k_o_) of 0.95 min^−1^. The superparamagnetic nature of Fe_3_O_4_ enabled facile recovery and reusability over multiple cycles without significant performance loss. This dual functionality—wastewater treatment and catalytic conversion—positions the rGO/Fe_3_O_4_/Ag nanocomposite as a versatile material for environmental remediation and industrial chemical synthesis.

## Data Availability

Data availability All data generated or analyzed during this study are included in this article.
